# Ecologically Valid Carbohydrate Intake during Soccer-Specific Exercise Does Not Affect Running Performance in a Fed State

**DOI:** 10.3390/nu9010039

**Published:** 2017-01-05

**Authors:** Mark P. Funnell, Nick R. Dykes, Elliot J. Owen, Stephen A. Mears, Ian Rollo, Lewis J. James

**Affiliations:** 1 School of Sport, Exercise and Health Sciences, Loughborough University, Leicestershire LE11 3TU, UK; M.Funnell2@lboro.ac.uk (M.P.F.); Nick.R.Dykes@gmail.com (N.R.D.); Elliot_Owen@hotmail.co.uk (E.J.O.); S.A.Mears@lboro.ac.uk (S.A.M.); 2 The Gatorade Sports Science Institute, PepsiCo Global Nutrition, Leicester LE3 9QH, UK; Ian.Rollo@Pepsico.com

**Keywords:** endurance, LIST, sports drink, ecological validity, sprinting

## Abstract

This study assessed the effect of carbohydrate intake on self-selected soccer-specific running performance. Sixteen male soccer players (age 23 ± 4 years; body mass 76.9 ± 7.2 kg; predicted VO_2max_ = 54.2 ± 2.9 mL∙kg^−1^∙min^−1^; soccer experience 13 ± 4 years) completed a progressive multistage fitness test, familiarisation trial and two experimental trials, involving a modified version of the Loughborough Intermittent Shuttle Test (LIST) to simulate a soccer match in a fed state. Subjects completed six 15 min blocks (two halves of 45 min) of intermittent shuttle running, with a 15-min half-time. Blocks 3 and 6, allowed self-selection of running speeds and sprint times, were assessed throughout. Subjects consumed 250 mL of either a 12% carbohydrate solution (CHO) or a non-caloric taste matched placebo (PLA) before and at half-time of the LIST. Sprint times were not different between trials (CHO 2.71 ± 0.15 s, PLA 2.70 ± 0.14 s; *p =* 0.202). Total distance covered in self-selected blocks (block 3: CHO 2.07 ± 0.06 km; PLA 2.09 ± 0.08 km; block 6: CHO 2.04 ± 0.09 km; PLA 2.06 ± 0.08 km; *p =* 0.122) was not different between trials. There was no difference between trials for distance covered (*p ≥* 0.297) or mean speed (*p ≥* 0.172) for jogging or cruising. Blood glucose concentration was greater (*p <* 0.001) at the end of half-time during the CHO trial. In conclusion, consumption of 250 mL of 12% CHO solution before and at half-time of a simulated soccer match does not affect self-selected running or sprint performance in a fed state.

## 1. Introduction

With sporting events being won and lost by the finest margins, athletes are constantly striving for ways to improve training and performance, and gain a competitive edge. One well-known method by which athletes can achieve this is through carbohydrate ingestion during prolonged (>1 h) bouts of exercise [1,2,3].

Fatigue during prolonged exercise is coupled with the depletion of glycogen stores [4], a decrease in blood glucose concentration [3], and a reduction in the rate of carbohydrate oxidation in the later stages of exercise [5]. Exogenous carbohydrate intake provides a method to significantly alter carbohydrate availability during exercise [6] and potentially offset the proposed factors that cause fatigue. The mechanisms include the prevention or reversal of hypoglycemia, a decrease in hepatic glucose output, and the maintenance of high rates of carbohydrate oxidation, especially in the later stages of exercise when endogenous glycogen stores are reduced [1,2,5].

A number of studies have reported a performance improvement of carbohydrate ingestion during prolonged continuous cycling [1,3] and running [7,8], as well as discontinuous, intermittent running [9,10,11]. More specifically, several studies have examined the effect of carbohydrate intake during simulated soccer or games performance, many implementing the Loughborough Intermittent Shuttle Test (LIST), a protocol specifically designed to replicate the demands of a soccer match [12]. Overall, these studies demonstrate that carbohydrate ingestion during exercise improves intermittent running capacity [10,11,13], and/or induces physiological alterations that promote improvements in performance [14,15,16]. Nicholas et al. [13] reported a 33% improvement in time to fatigue (alternating between 20 m shuttles of jogging and cruising) following 75 min of the LIST when subjects consumed a 6.9% carbohydrate solution before and every 15 min throughout exercise. Similarly, Davis et al. [17] found a 32% increase in intermittent running time to fatigue when a 6% carbohydrate solution was consumed before and every 15 min throughout the LIST.

Despite a number of studies reporting improvements on simulated soccer performance, many have methodological limitations that restrict their application to real-world soccer match performance. Several studies have not used ecologically valid feeding strategies, for example testing subjects in a fasted state [13,15,17], or providing carbohydrate drinks regularly throughout exercise [10,13,18]. Typically, players would not begin a soccer match in a fasted state, and apart from extenuating circumstances (i.e., a severe injury or excessively hot environmental conditions) would not be able to drink during each 45 min half of soccer. Additionally, the validity of adopting an exhaustive protocol at the end of simulated soccer match [10,13,17] needs to be questioned, as soccer players are rarely required to run until exhaustion at the end of competition.

Therefore, the purpose of this study was to investigate the effect of carbohydrate intake in a fed state, using an ecologically valid feeding strategy (i.e., before and at half-time of a simulated soccer match), on sprint and self-selected soccer-specific running performance in competitive soccer players.

## 2. Materials and Methods

### 2.1. Subjects

Sixteen male soccer players (age 23 ± 4 years; body mass 76.9 ± 7.2 kg; height 1.81 ± 0.06 m; body mass index 23.5 ± 1.9 kg∙m^−2^; predicted VO_2max_ 54.2 ± 2.9 mL∙kg^−1^∙min^−1^; body fat 11.8% ± 3.0%) volunteered for this study, which was approved by the Loughborough University Ethics Approvals (Human Participants) Sub Committee (reference number: R15-P159). Before commencement of the study, subjects provided written informed consent and completed a medical screening questionnaire. Subjects were non-smokers, had a soccer playing history of 13 ± 4 years, and participated in soccer training and/or matches 3 ± 1 times per week. All subjects completed a preliminary trial, familiarisation trial and two experimental trials, separated by a minimum of 3 days.

### 2.2. Pre-Trial Standardisation

To ensure similar metabolic conditions prior to each experimental trial, all subjects recorded their dietary intake and habitual physical activity for the day preceding and day of their first experimental trial. These diet and activity patterns were replicated prior to the second experimental trial and adherence to these requirements was verbally checked. Subjects also refrained from any strenuous exercise or alcohol intake during this period. The specific time of trials was standardised for each subject to prevent any diurnal effects. Subjects consumed a standardised pre-trial meal providing 2 g∙carbohydrate∙kg^−1^ body mass (BM) (consisting of Nutri-grain cereal bars and semi-skimmed milk at a ratio of 30 g cereal bar to 125 g milk) 2.5 h before arrival at the laboratory, and consumed 500 mL of water 1.5 h before arrival to the laboratory (i.e., ~3 h and ~2 h pre-exercise, respectively).

### 2.3. Preliminary Testing

During the first visit, body mass and height (Model 285, Seca Scales, Birmingham, UK) were recorded, and body fat percentage was estimated using skinfold caliper measurements at four sites (i.e., biceps, triceps, sub-scapular and supra-iliac [19]). Each subject’s VO_2max_ and velocity at VO_2max_ were predicted using a progressive multistage shuttle run test [20], and these data were used to determine the timing intervals for jogging (55% VO_2max_) and cruising (95% VO_2max_) during the experimental trials. All trials were completed on an indoor 20 m running track, with only one subject completing the test at a time.

### 2.4. Experimental Trials

**Figure 1:**
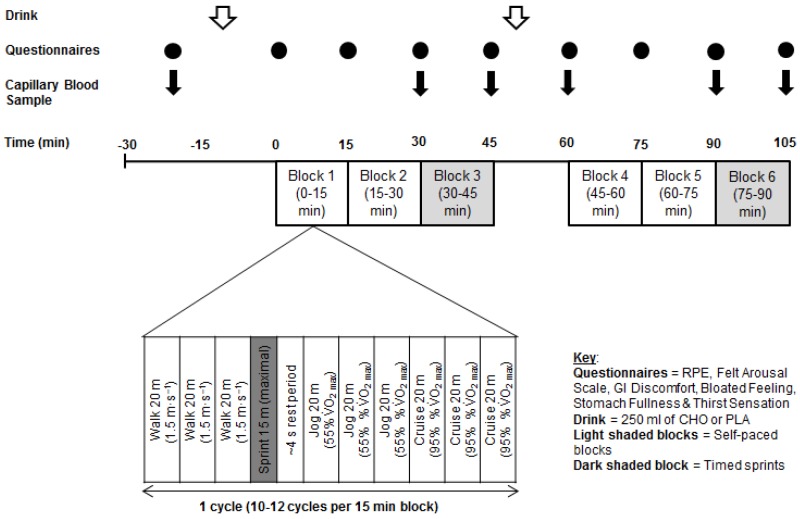
Schematic of the study design.

Timing of trials was standardised within subjects. Upon arrival at the laboratory, subjects voided their bladder into a plastic container, and nude body mass was recorded. This urine sample was analysed for osmolality (Osmocheck, Vitech Scientific, Horsham, UK) to assess hydration status, and a urine osmolality of <900 mOsm∙kgH_2_O^−1^ was taken to indicate the absence of hypohydration [21]. No subject produced a urine sample above this value. After 10 min of seated rest, heart rate was recorded and a finger-prick capillary blood sample was taken. Subjects then completed a 10 min self-selected warm-up, which they were asked to replicate during both experimental trials. Thereafter, subjects drank 250 mL of test drink over a 5 min period before resting for a further 5 min. Subjects then began a modified version of the LIST (Figure 1).

During the modified LIST, subjects were required to walk and run back and forth between two lines 20 m apart. Each block of the LIST was 15 min in duration and consisted of ~11 repeated cycles of walking (three shuttles at 1.5 m∙s^−1^), sprinting (15 m), rest (4 s), jogging (three shuttles at 55% predicted VO_2max_) and cruising (three shuttles at 95% predicted VO_2max_). Subjects completed six blocks of the LIST totalling 90 min of simulated soccer performance. A 15 min passive recovery period (i.e., half-time) separated the six blocks of the LIST into two 45 min halves. The exercise intensities throughout the first two blocks of both halves (blocks 1–2 and 4–5; 0–30 min) were dictated by audio cues as per the LIST protocol [12]. The third block in each half (blocks 3 and 6; 30–45 min) was modified by removing the audio-cues and was therefore “self-selected” [22]. During the self-selected blocks, subjects were instructed to replicate the patterns and intensities of the audio cue dictated blocks, but were provided with no cues or other verbal instructions [22]. Subjects were video recorded during the self-selected blocks, and analysis of the video provided a record of the time and distance spent in each activity. Sprint times were recorded during all six blocks of the LIST over 15 m using infrared timing gates (Brower Timing Systems, Draper, UT, USA), with the timing gate height standardised at 24.5 cm. Subjects drank a further 250 mL of test drink during the first five min of half-time, with water consumed ad-libitum for the remainder of the half-time break of the familiarisation trial (i.e., 5–15 min). Subjects replicated this water ingestion during experimental trials for standardisation purposes. Upon completion of the LIST, subjects had a 10-min period to cool down, before nude body mass was measured to determine change in body mass.

Heart rate (M400, Polar Electro, Kempele, Finland) was recorded continuously throughout exercise and averaged for each 15 min block of the LIST. Finger-prick capillary blood samples (20 µL) were taken at rest, immediately before and after each self-selected block, and at the end of half-time, and were analysed for blood glucose and lactate (Biosen EKF Diagnostic, HaB Direct, Warwickshire, UK). Arousal (Felt Arousal Scale [23]), GI discomfort, bloated feeling, stomach fullness and thirst sensation were recorded at rest and every 15 min throughout exercise. GI discomfort, bloated feeling, stomach fullness and thirst sensation were scored on a 10-point scale (1 = not at all, 10 = very, very much [24]). Rating of perceived exertion (RPE [25]) was recorded every 15 min throughout exercise.

### 2.5. Test Drinks

Subjects were given either a carbohydrate (CHO) or placebo (PLA) drink in a double-blind, randomised, counter-balanced design. The CHO drink was a 12% solution delivering 60 g carbohydrate per 500 mL bottle of a blend of sucrose, maltodextrin and isomaltulose, whilst the PLA drink was non-caloric and taste matched to the CHO drink using artificial sweeteners (sucralose and acesulfame potassium). Both drinks contained matched amounts of sodium (18 mmol∙L^−1^) and potassium (4 mmol∙L^−1^).

### 2.6. Statistical Analysis

Data were analysed using SPSS (version 22, SPSS Inc., Chicago, IL, USA) and were initially checked for normality of distribution using a Shapiro–Wilk test. Pre-trial measures were analysed using paired *t*-tests or Wilcoxon Signed Rank tests, as appropriate. Running performance data, blood glucose, blood lactate, heart rate, mean sprint times and subjective feelings questionnaires were analysed using a two-way (time × drink) repeated measures ANOVA. Where the assumption of sphericity was violated, the degrees of freedom were corrected using the Greenhouse–Geisser estimate. Significant results were followed-up by post-hoc paired *t*-tests or Wilcoxon Signed Rank tests, as appropriate, and the familywise error rate was controlled using the Holm–Bonferroni adjustment. Data sets were accepted as being significantly different when *p ≤* 0.05. All data are presented as mean ± SD, unless stated otherwise.

## 3. Results

### 3.1. Pre-Trial Measures

Pre-trial urine osmolality (CHO 400 ± 240 mOsm∙kgH_2_O^−1^, PLA 386 ± 257 mOsm∙kgH_2_O^−1^; *p =* 0.828) and body mass (CHO 76.4 ± 7.0 kg, PLA 76.7 ± 6.9 kg; *p =* 0.195) were not different between trials.

### 3.2. Running Performance

There was no trial order effect, with similar distances completed for block 3 (trial 1: 2.09 ± 0.07 km, trial 2: 2.07 ± 0.07 km; *p =* 0.305) and block 6 (trial 1: 2.06 ± 0.09 km, trial 2: 2.04 ± 0.07 km, *p =* 0.114) and mean running speeds for block 3 (trial 1: 8.3 ± 0.3 km∙h^−1^, trial 2: 8.3 ± 0.3 km∙h^−1^; *p =* 0.305) and block 6 (trial 1: 8.3 ± 0.4 km∙h^−1^, trial 2: 8.1 ± 0.3 km∙h^−1^; *p =* 0.114) during the first and second experimental trials.

**Figure 2:**
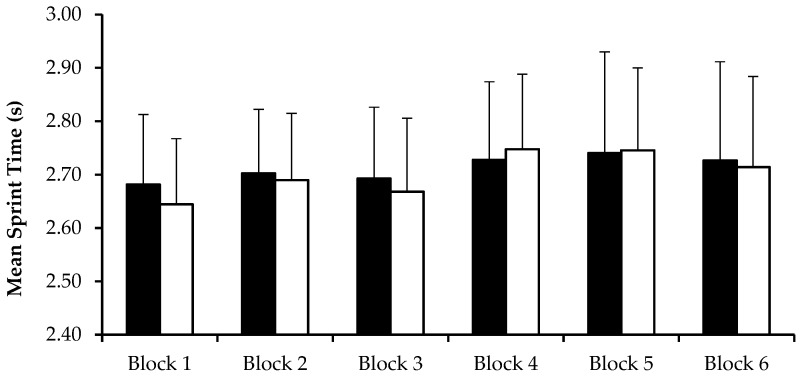
Mean sprint times for the six blocks of the LIST during CHO (■) and PLA (□) trials. LIST: Loughborough Intermittent Shuttle Test; CHO: carbohydrate trial; PLA: placebo trial.

There was no difference (*p =* 0.202) in 15 m sprint times between CHO and PLA trials during any of the six blocks of the LIST (Figure 2). There was a main effect of time (*p =* 0.013), with faster 15 m sprint times (*p <* 0.001) during the first three blocks (2.68 ± 0.13 s) compared to the final three blocks of the LIST (2.73 ± 0.16 s). There was no time by trial interaction effect (*p =* 0.337) for 15 m sprint times.

The self-selected distance covered and mean speed for blocks 3 and 6 of the LIST during the CHO and PLA trials are displayed in Table 1. There were no differences in total distance covered (*p =* 0.122) or mean speed (*p =* 0.070) between CHO or PLA trials. There was a main effect of time for total distance covered (*p =* 0.011) and mean speed (*p =* 0.005), with more distance covered and faster mean speeds in block 3 (distance 2.08 ± 0.07 km; speed 8.3 ± 0.3 km∙h^−1^) than block 6 (distance 2.05 ± 0.08 km; speed 8.2 ± 0.3 km∙h^−1^). There were no differences in distance covered (*p ≥* 0.297) or mean speed (*p ≥* 0.172) for jogging and cruising between CHO and PLA trials. There was a main effect of trial for both distance covered (*p =* 0.030) and mean speed (*p =* 0.045) for walking, however, post-hoc tests revealed no differences during block 3 (distance *p =* 0.232; mean speed *p =* 0.246) or block 6 (distance *p =* 0.370; mean speed *p =* 0.098). There were no interaction effects (*p ≥* 0.343) for total distance covered or mean speed, or distance covered and mean speed for walking, jogging or cruising. There were also no time (*p ≥* 0.497), trial (*p ≥* 0.131) or interaction (*p ≥* 0.905) effects for total recovery time or mean recovery time per lap of the LIST.

### 3.3. Subjective Feelings Questionnaires

**Figure 3:**
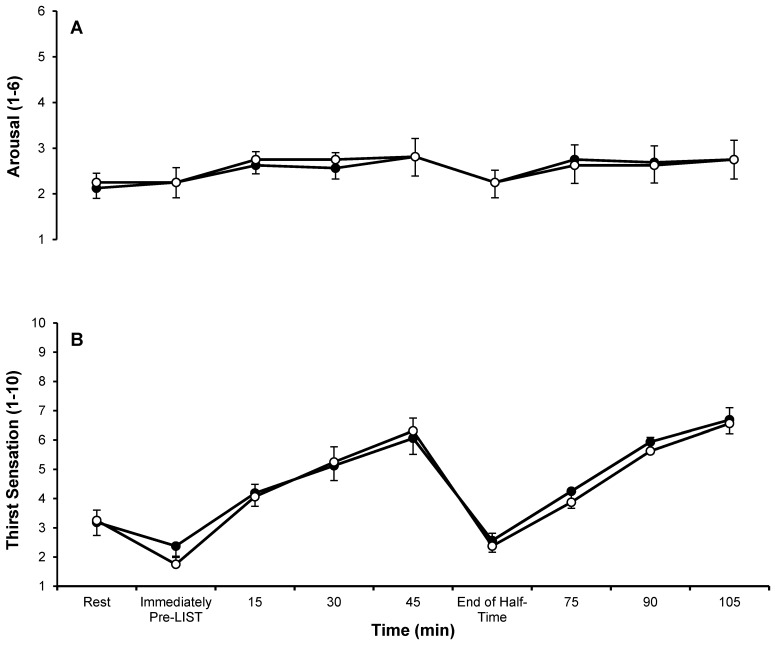
Subjective feelings questionnaires for (**A**) arousal (1 = low arousal; 6 = high arousal) and (**B**) thirst sensation (1 = not at all; 10 = very, very much) during CHO (●) and PLA (○) trials. Data are mean ± SE. CHO: carbohydrate trial; PLA placebo trial.

**Figure 4:**
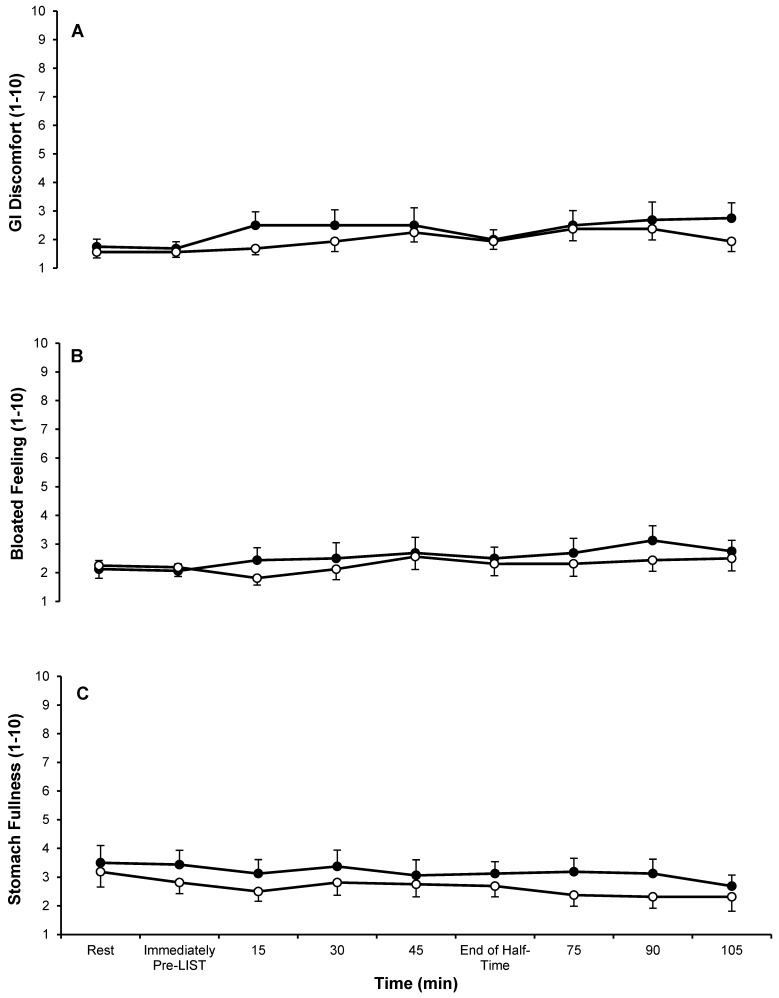
Subjective feelings questionnaires for (**A**) GI discomfort; (**B**) bloated feeling and (**C**) stomach fullness (A, B, C: 1 = not at all; 10 = very, very much) during CHO (●) and PLA (○) trials. Data are mean ± SE. GI discomfort: gastrointestinal discomfort; CHO: carbohydrate trial; PLA placebo trial.

Data from the subjective feelings questionnaires are displayed in Figure 3 and Figure 4. There were main effects of time for thirst sensation (*p <* 0.001) and GI discomfort (*p =* 0.026), but not for arousal (*p =* 0.198), stomach fullness (*p =* 0.409) or bloating feeling (*p =* 0.248). There was a main effect of trial for stomach fullness (*p =* 0.001); with subjects reporting higher levels of stomach fullness during the CHO trial (3.2 ± 1.9) compared to the PLA trial (2.6 ± 1.7), but no interaction effect (*p =* 0.759). There were no main effects of trial (GI discomfort *p =* 0.091; bloated feeling *p =* 0.235; arousal *p =* 0.805; thirst sensation *p =* 0.520) or interaction effects (GI discomfort *p =* 0.477; bloated feeling *p =* 0.547; arousal *p =* 0.831; thirst sensation *p =* 0.337) for any of the other subjective feelings questionnaires.

### 3.4. Physiological Measures

Due to problems with blood collection during two experimental trials, the blood glucose and lactate analysis only includes 14 subjects. There were main effects of time for both blood glucose and lactate concentration (*p <* 0.001; Table 2). There was an interaction effect for blood glucose concentration (*p <* 0.001), with greater blood glucose concentration at the end of half-time during the CHO trial (CHO 6.6 ± 0.7 mmol∙L^−1^; PLA 5.0 ± 0.7 mmol∙L^−1^; *p <* 0.001). There were no main effects of trial for blood glucose (*p =* 0.265) or lactate (*p =* 0.637) concentrations and no interaction effect for blood lactate concentration (*p =* 0.399).

Ad-libitum fluid intake during half-time of the familiarisation trial was 238 ± 162 mL and all subjects replicated this volume of fluid intake in the experimental trials. There was no difference in the percentage of body mass lost between trials (CHO 1.67% ± 0.49%, PLA 1.76% ± 0.66%; *p =* 0.087), or total sweat loss between trials (CHO 1.94 ± 0.32 L, PLA 1.99 ± 0.40 L; *p =* 0.310).

There were no trial (heart rate *p =* 0.626; RPE *p =* 0.503) or interaction (heart rate *p =* 0.228; RPE *p =* 0.511) effects for heart rate or RPE (Table 3). There was a main effect of time (*p <* 0.001) for heart rate and RPE. Heart rate increased from rest to block 1 of the LIST and remained elevated throughout exercise, while RPE increased gradually throughout exercise.

### 3.5. Perception of Test Drinks

Upon completion of the second experimental trial, subjects were asked if they could distinguish between the test drinks, and if so, which of the trials they believed was the CHO drink. Of the 16 subjects recruited, 10 believed they could distinguish between the two test drinks; with seven of these subjects correctly identifying the CHO drink.

## 4. Discussion

The purpose of the present study was to assess the effect of carbohydrate intake using an ecologically valid feeding strategy (i.e., before and at half-time) on sprint and self-selected soccer-specific running performance in a fed state. The main finding was that consumption of 250 mL of a 12% carbohydrate solution (40 g∙h^−1^) before and at half-time of a simulated soccer match did not improve self-selected running performance or sprint speed. Despite the lack of effect of carbohydrate intake on running performance, the current study provides clarity in an area of the literature where the majority of studies are limited by ecological validity [10,13,15,17].

The main finding is in contrast to a number of similar studies that have examined the effect of carbohydrate intake on simulated soccer performance [10,13,15,17]. Nicholas et al. [13] reported a 33% improvement in running time to fatigue (alternating between 20 m shuttles of jogging and cruising) following 75 min of the LIST when subjects consumed a 6.9% carbohydrate solution before (5 mL∙kg^−1^ BM) and every 15 min throughout exercise (2 mL∙kg^−1^ BM). Likewise, Davis et al. [17] reported a 32% increase in intermittent running time to fatigue when a 6% carbohydrate solution was consumed before (5 mL∙kg^−1^ BM) and every 15 min throughout the LIST (2 mL∙kg^−1^ BM). Both of these studies, like several others that have found beneficial effects of carbohydrate ingestion on intermittent running performance [10,11,26], implemented a time to fatigue protocol at the end of a prolonged period of intermittent running. The validity of implementing an exhaustive protocol needs to be questioned, as soccer players are rarely required to run until fatigue at the end of a match. In this study, the self-paced block at the end of each half, which followed two prescribed blocks of intermittent exercise, allowed quantification of intermittent running performance when fatigue is normally present during a soccer match [22], and did so in a more ecologically valid manner [26]. 

Moreover, in the present study, subjects consumed a pre-trial meal consistent with pre-exercise guidelines; i.e., 1–4 g·carbohydrate∙kg^−1^ BM in the 4 h before exercise [27]. The majority of studies that have found a positive effect of carbohydrate ingestion on intermittent running performance required subjects to arrive at the laboratory after an overnight fast (8–10 h) [11,13,17]. Conversely, one study [18] asked subjects to mimic their normal pre-match nutrition practices, and found no benefit of ingestion of a 7% carbohydrate solution on intermittent running time to fatigue following 75 min of the LIST. It is important to note that a lower rate of carbohydrate ingestion of 33 g∙h^−1^ was used by Goedecke et al. [18], which is towards the lower end of the recommended amount of 30–60 g∙h^−1^ for prolonged exercise [28]. In other exercise modes the importance of a pre-trial meal has been shown. Sherman et al. [29] found ~45 min cycling time trial performance after a 2 h preload was improved following a low- (1.1 g∙kg^−1^ BM) and high- (2.2 g∙kg^−1^ BM) carbohydrate pre-trial beverage compared to a non-caloric placebo following an overnight fast. The pre-trial meal implemented in the present study may have negated the beneficial effects of carbohydrate ingestion during exercise, potentially explaining the absence of an effect of carbohydrate ingestion on self-paced running or sprint performance. It could also explain the presence of a beneficial effect of carbohydrate intake in other studies that required subjects to exercise under fasted conditions [13,17].

Another possible reason for the different outcome in the present study compared to other studies that have investigated carbohydrate intake on intermittent running performance could be the regularity of carbohydrate ingestion throughout exercise. Typically, players would not be able to consume fluids or food during each 45 min half of soccer unless extenuating circumstances (i.e., a severe injury or excessively hot environmental conditions) permitted. In this study, subjects consumed 250 mL of carbohydrate solution before and at half-time, whereas in other similar studies [10,13,17,18] subjects ingested carbohydrate before, every 15 min throughout exercise, and at half-time. Despite the less frequent ingestion in this study, subjects consumed a total of 60 g of carbohydrate, 30 g before exercise and 30 g at half-time, equating to an ingestion rate of 40 g∙h^−1^. Although this rate of carbohydrate ingestion is in alignment with the recommended amount for prolonged (1–2 h) exercise of 30–60 g∙h^−1^ [28], no differences in sprint or self-paced running performance were found between trials.

Evidence is inconsistent with regards to the effect of carbohydrate ingestion on sprint performance during intermittent running exercise [26,30]. Nicholas et al. [13] found no difference in 15 m sprint times with ingestion of a 6.9% carbohydrate solution compared to a placebo solution during 75 min of the LIST. Likewise, Foskett et al. [9] reported no improvement in 15 m sprint times when a 6.4% carbohydrate solution was consumed at a high rate of 90 g∙h^−1^, compared to a placebo, during 90 min of the LIST. Interestingly, Foskett et al. [9] found no glycogen sparing effect of carbohydrate ingestion during 90 min of the LIST when subjects began exercise with high pre-exercise muscle glycogen concentrations. The results of the present study demonstrate that ingestion of a 12% carbohydrate solution had no effect on 15 m sprint times during any of the six blocks of the LIST. This finding is in agreement with several studies that have used similar protocols to replicate the demands of soccer [9,13,14,16].

Conversely, there are a few studies that have reported an improvement in sprint performance [11,15]. Welsh et al. [11] investigated the effect of a high rate of carbohydrate ingestion before (5 mL∙kg^−1^ BM of a 6% carbohydrate solution), in between quarters (3 mL∙kg^−1^ BM of a 6% carbohydrate solution) and at half-time (5 mL∙kg^−1^ BM of a 18% carbohydrate solution) on 20 m sprint times during an intermittent high-intensity shuttle running protocol consisting of 15-min quarters. Sprint times were ~14% faster in the fourth quarter when subjects consumed carbohydrate. Ali et al. [15] found 15 m sprint times were 1.2% faster during 90 min of the LIST when subjects consumed a 6.4% carbohydrate solution compared to a placebo solution. However, a glycogen depleting bout of exercise was completed the day before experimental trials and subjects began trials after an overnight fast. Therefore, endogenous glycogen stores would have been sub-optimal, and it is likely that the effects of carbohydrate ingestion on exercise performance would be augmented in this state. As muscle glycogen is an important substrate for high-intensity efforts during intermittent running exercise [30], it could be speculated that the effectiveness of carbohydrate ingestion on sprint performance may largely depend on endogenous muscle glycogen concentrations [26].

Nicholas et al. [14] found ingestion of a 6.9% carbohydrate solution during 90 min of the LIST resulted in a 22% reduction in muscle glycogen utilisation compared to a placebo solution. Similarly, a 24% reduction in muscle glycogen utilisation was observed by Tsintzas et al. [4] at the point of exhaustion during a placebo trial compared to a carbohydrate trial (5.5% carbohydrate solution before and every 20 min during exercise), when subjects were required to run until exhaustion at 70% VO_2max_. While muscle glycogen content was not measured in the present study, if the ingestion of carbohydrate before and at half-time did reduce the utilisation of muscle glycogen, the absence of an effect on self-selected running or sprint performance between trials suggests the duration and/or nature of the exercise did not deplete glycogen stores significantly enough to impact on performance. However, both Nicholas et al. [14] and Tsintzas et al. [4], performed trials after an overnight fast, meaning liver glycogen would have been sub-optimal compared to a fed state [31]. Consumption of a high carbohydrate breakfast (2.5 g∙carbohydrate∙kg^−1^ BM) has been shown to increase muscle glycogen concentrations by 11% three hours post ingestion [32]. The pre-trial meal, consisting of 2 g∙carbohydrate∙kg^−1^ BM, consumed ~3 h before exercise in the present study may have replenished liver glycogen and elevated muscle glycogen concentrations, potentially negating a beneficial effect of carbohydrate ingestion during exercise.

It is well known that carbohydrate ingestion during exercise alters blood glucose concentration either intermittently or throughout exercise [1,2,9,13,15]. The only observed difference in blood metabolites in this study was an elevated blood glucose concentration at the end of half-time during the CHO trial (Table 2). Despite this difference, self-selected running and sprint performance were similar between trials. Importantly, blood glucose concentration did not reach hypoglycemia in either trial, indicating that the endogenous glycogen stores and the pre-trial meal consumed were sufficient to maintain blood glucose concentration throughout 90 min of the LIST protocol [27].

The carbohydrate concentration (12%) of the solution administered in the present study was considerably greater than the 6%–7% carbohydrate concentration adopted by many other similar studies [13,17,18]. Despite this, there were very few incidences of gastro-intestinal (GI) discomfort, or adverse subjective ratings of bloated feeling or stomach fullness in either trial (Figure 4), with only one subject reporting GI distress. It is important to note that this subject reported relatively high GI distress in the PLA trial (GI discomfort: 4 ± 1; bloated feeling: 5 ± 2; stomach fullness: 5 ± 2) as well as the CHO trial (GI discomfort: 6 ± 3; bloated feeling: 6 ± 3; stomach fullness: 6 ± 3), suggesting that the ingestion of fluid, and not the carbohydrate content may have been the cause. The low volume of fluid consumed (250 mL before and at half-time) and the fact that the subjects were familiar with consuming fluid and/or commercially available carbohydrate-electrolyte drinks throughout soccer training and matches may help to explain the low incidence of subjective ratings of GI discomfort, bloated feeling or stomach fullness.

## 5. Conclusions

In conclusion, consumption of 250 mL of a 12% carbohydrate solution before and at half-time of a simulated soccer match does not affect self-selected running performance or sprint times in a fed state.

## Figures and Tables

**Table 1:** Self-selected running performance for blocks 3 and 6 of the LIST during CHO and PLA trials.

Variable	Block 3		Block 6	
	CHO	PLA	CHO	PLA
Total				
Distance Covered (km)	2.07 ± 0.06	2.09 ± 0.08	2.04 ± 0.09	2.06 ± 0.08
Mean Speed (km∙h^−1^)	8.3 ± 0.3	8.3 ± 0.3	8.1 ± 0.3	8.2 ± 0.3
Walking				
Distance Covered (km)	0.66 ± 0.02	0.67 ± 0.02	0.65 ± 0.02	0.66 ± 0.03
Mean Speed (km∙h^−1^)	5.6 ± 0.2	5.7 ± 0.2	5.5 ± 0.2	5.6 ± 0.2
15 m Sprint				
Distance Covered (m)	161 ± 7	161 ± 7	160 ± 7	160 ± 7
Mean Sprint Time (s)	2.69 ± 0.13	2.67 ± 0.14	2.73 ± 0.18	2.71 ± 0.17
Mean Speed (km∙h^−1^)	20.1 ± 1.0	20.3 ± 1.1	19.9 ± 1.3	20.0 ± 1.2
Jogging				
Distance Covered (km)	0.63 ± 0.03	0.63 ± 0.03	0.62 ± 0.04	0.63 ± 0.03
Mean Speed (km∙h^−1^)	10.9 ± 0.6	11.0 ± 0.7	10.7 ± 0.8	10.8 ± 0.7
Cruising				
Distance Covered (km)	0.62 ± 0.03	0.62 ± 0.03	0.60 ± 0.03	0.61 ± 0.03
Mean Speed (km∙h^−1^)	12.7 ± 0.4	12.9 ± 0.5	12.7 ± 0.4	12.7 ± 0.6
Recovery Time				
Total (s)	40 ± 5	38 ± 7	41 ± 7	39 ± 7
Mean Per Lap (s)	4 ± 0	4 ± 1	4 ± 1	4 ± 1

Data are mean ± SD. CHO: carbohydrate trial; PLA: placebo trial.

**Table 2:** Blood glucose and lactate concentrations (mmol∙L^−1^) during CHO and PLA trials.

	Rest	30 min	45 min	End of Half-Time	90 min	105 min
**Blood glucose (mmol∙L^−1^)**						
CHO	4.8 ± 0.8	5.0 ± 0.6	5.1 ± 0.6	6.6 ± 0.7 ^†^	4.1 ± 0.6	4.7 ± 0.6
PLA	5.2 ± 0.5	4.8 ± 0.6	5.1 ± 0.4	5.0 ± 0.7	4.6 ± 0.5	4.8 ± 0.6
**Blood lactate (mmol∙L^−1^)**						
CHO	1.3 ± 0.2	3.5 ± 1.5	3.6 ±1.5	2.4 ± 0.9	3.4 ± 1.5	3.4 ± 1.4
PLA	1.3 ± 0.4	3.8 ± 1.6	3.7 ± 1.6	2.2 ± 0.9	3.1 ± 1.4	3.1 ± 1.4

^†^ Indicates significantly (*p <* 0.05) different between trials. Data are mean ± SD; *n =* 14. CHO: carbohydrate trial; PLA placebo trial.

**Table 3:** Heart rate (beat∙min^−1^) and RPE (6–20) during the LIST for CHO and PLA trials.

	Rest	Block 1	Block 2	Block 3	Block 4	Block 5	Block 6
**Heart rate (beat∙min^−1^)**							
CHO	63 ± 10	154 ± 11	166 ± 11	164 ± 10	157 ± 10	167 ± 11	162 ± 13
PLA	62 ± 10	154 ± 10	166 ± 11	165 ± 10	156 ± 10	164 ± 10	163 ± 11
**RPE (6–20)**							
CHO	-	13 ± 2	14 ± 2	14 ± 2	14 ± 2	15 ± 2	16 ± 2
PLA	-	13 ± 2	14 ± 2	15 ± 2	14 ± 2	15 ± 2	16 ± 2

Data are mean ± SD. RPE: rating of perceived exertion; CHO: carbohydrate trial; PLA placebo trial.
